# Coptisine, a protoberberine alkaloid, relaxes mouse airway smooth muscle via blockade of VDLCCs and NSCCs

**DOI:** 10.1042/BSR20190534

**Published:** 2020-02-25

**Authors:** Nana Wen, Lu Xue, Yongle Yang, Shunbo Shi, Qing-Hua Liu, Congli Cai, Jinhua Shen

**Affiliations:** 1 Institute For Medical Biology and Hubei Provincial Key Laboratory for Protection and Application of Special Plants in Wuling Area of China, College of Life Sciences, South-Central University for Nationalities, Wuhan 430074, China; 2 Wuhan Youzhiyou Biopharmaceutical Co., Ltd., 666 Gaoxin Rd, Biolake, Wuhan 430075, China

**Keywords:** ASM, asthma, coptisine, NSCCs, VDLCCs

## Abstract

***Background/Aims:*** Recently, effective and purified ingredients of traditional Chinese medicine (TCM) were extracted to play crucial roles in the treatment of pulmonary diseases. Our previous research focused on TCM drug screening aimed at abnormal airway muscle contraction during respiratory diseases. Coptisine, an effective ingredient extracted from bitter herbs has shown a series of antioxidant, antibacterial, cardioprotective and neuroprotective pharmacological properties. In the current study, we questioned whether coptisine could also participate in asthma treatment through relaxing abnormal contracted mouse airway smooth muscle (ASM). The present study aimed to characterize the relaxant effects of coptisine on mouse ASM and uncover the underlying molecular mechanisms. ***Methods:*** To investigate the role of coptisine on pre-contracted mouse ASM, a series of biological techniques, including force measurement and patch-clamp experiments were employed. ***Results:*** Coptisine was found to inhibit high K^+^ or acetylcholine chloride (ACh)-induced pre-contracted mouse tracheal rings in a dose-dependent manner. Further research demonstrated that the coptisine-induced mouse ASM relaxation was mediated by alteration of calcium mobilization via voltage-dependent L-type Ca^2+^ channels (VDLCCs) and non-selective cation channels (NSCCs). ***Conclusion:*** Our data showed that mouse ASM could be relaxed by coptisine via altering the intracellular Ca^2+^ concentration through blocking VDLCCs and NSCCs, which suggested that this pharmacological active constituent might be classified as a potential new drug for the treatment of abnormal airway muscle contraction.

## Introduction

Pulmonary diseases are a series of debilitating, life-threatening respiratory illnesses that have become severe worldwide public health problems and financial burdens [1]_._ According to a recent study, asthma and chronic obstructive pulmonary disease (COPD) together threatened 300 million people worldwide [2]. Airway inflammation, excessive cell matrix proliferation, especially the abnormal contraction of airway smooth muscle (ASM) are the main symptoms of pulmonary diseases [3–5]. The development of effective medications for pulmonary diseases and improving quality of life without side effects are urgently needed.

As well known, traditional Chinese medicine (TCM) especially a large number of herbal formulations play important roles in pulmonary diseases treatment [6]. However, rigorous Western methodologies should be employed to isolate the effective substances from the complex mixture of chemicals for scientific validation and further clinical application. Our previous studies have revealed that quite a few effective ingredients or extracts of TCM could relax abnormal smooth muscle contraction in pulmonary diseases [7,8].

Coptisine, a protoberberine alkaloid (PBA), is an active constituent which has been identified in a lot of Chinese herbs, especially in *Coptis* species [9], such as *C. teeta* and *C. chinensis*. Coptisine has also been characterized in other plant species such as *Coptidis rhizoma, Epimedium sagittatum* [10], *Chelidonium majus* L. [11] and etc. In all these studies, coptisine has exhibited antibacterial [12], antioxidant [13], cardioprotective [14], neuroprotective [15] antispasmodic and relaxant [11] properties. Nonetheless, research about coptisine aimed at ameliorating excessive abnormal contraction in ASM, which is a key symptom of pulmonary disease, has been limited.

The purpose of present study was to investigate the roles of coptisine in pulmonary disease treatments with a focus on mouse ASM relaxation. The results showed that acetylcholine chloride (ACh) or high K^+^ precontracted mouse ASM could be relaxed by coptisine in a concentration-dependent manner. Further research indicated that coptisine exerted its relaxant effects by decreasing intracellular calcium via voltage-dependent L-type Ca^2+^ channels (VDLCCs) and non-selective cation channels (NSCCs).

## Materials and methods

### Reagents and chemicals

Coptisine with a purity >0.98 was purchased from ESITE Biotech Co. Ltd., (Chengdu, China) and dissolved in dimethyl sulfoxide (DMSO) for use. ACh, gadolinium, pyrazole 3 (Pyr3), nifedipine, niflumic acid (NA) and tetraethylammonium chloride (TEA), KB-R7943 were purchased from Sigma Chemical Co. (St. Louis, MO, U.S.A.). All other reagents were of analytical purity and purchased from Sinopharm Chemical Reagent Co. (Shanghai, China).

### Animals

All the animal experiments were designed and performed as previously described [7]. To be brief, sexually mature male BALB/c mice were purchased from the Hubei Provincial Center for Disease Control and Prevention (Wuhan, China). The mice were housed in a specific pathogen-free (SPF)-grade laboratory under a 12-h light–dark cycle. All animal experiments were approved by the Animal Care and Ethics Committee of the South-Central University for Nationalities (Wuhan, China) and were performed under the supervision of the Institutional Animal Care and Use Committee of the South-Central University for Nationalities (Wuhan, China).

### ASM contraction measurement

The tension of mouse tracheal ring was measured isometrically as previously described [16]. The mice were killed by cervical dislocation and the tracheal rings (5–7 mm) were cut and quickly transferred to ice-cold physiological salts solution (PSS) (in mM: NaCl 135, KCl 5, MgCl_2_ 1, CaCl_2_ 2, HEPES 10, glucose 10, pH 7.4) or Li-PSS (in mM: LiCl 135, KCl 5, MgCl_2_ 1, CaCl_2_ 2, HEPES 10, glucose 10, pH 7.4) without sodium. Each tracheal ring was mounted with a preload of 0.5 g in a 10-ml organ bath containing PSS or Li-PSS gassed continuously with a 95% O_2_ and 5% CO_2_ mixture at 37°C. After an initial 60-min equilibration, tracheal rings were given a successive stimulation with either high K^+^ (80 mM) or ACh (100 μM). To obtain the concentration–response curves, coptisine (0.01–1000 μM) was added cumulatively to the pre-contracted tracheal rings. Particular channel inhibitors including nifedipine (10 μM), Pyr3 (30 μM), gadolinium (30 μM), TEA (10 mM), KB-R7943 (10 μM) and etc were applied in ASM contraction measurements, respectively. To clarify the role of calcium in coptisine-induced contraction, the experiments were carried out in Ca^2+^-free PSS solution (0 mM Ca^2+^ and 0.5 mM EGTA).

### Isolation of ASM cells

Mouse ASM cells were isolated as described previously [17–19]. Briefly, tracheas were freshly isolated and digested in ASM dissociation buffer (in mM: NaCl 136, KCl 5.36, KH_2_PO_4_ 0.44, NaHCO_3_ 4.16, Na_2_HPO_4_.12H_2_O 0.34, HEPES 20, glucose 10, pH 7.1) containing 3 mg/ml papain, 0.15 mg/ml dithioerythritol and 1 mg/ml bovine serum albumin (BSA) at 35°C for 22 min. Then the digested tissues were transferred to ASM dissociation buffer containing 1 mg/ml collagenase H, and 1 mg/ml BSA and were incubated at 35°C for 8 min. The tissues were washed and gently triturated with 1 mg/ml BSA to yield single ASM cells for use in subsequent experiments.

### Measurement of VDLCC and NSCC currents

The VDLCC currents were measured using an EPC-10 patch-clamp amplifier (HEKA, Lambrecht, Germany) as previously described [16]. Ba^2+^ was employed as a charge carrier. The pipette was filled with intracellular solution (in mM: CsCl 130, EGTA 10, MgCl_2_ 4, Mg-ATP 4, HEPES 10, TEA 10, pH 7.2). ASM cells were patched and held in the bath solution (in mM: NaCl 107, BaCl_2_ 27.5, HEPES 10, glucose 11, TEA 10, pH 7.4) at −70 mV. Currents were measured following depolarization for 500 ms from −70 to +40 mV in 10 mV increments every 50 ms.

For the measurement of NSCC currents, the pipette was filled with solution (in mM: CsCl 126, MgCl_2_ 1.2, HEPES 10, EGTA 3 and CaCl_2_ 1, pH 7.2). The bath solution (in mM: NaCl 126, CaCl_2_ 1.5, HEPES 10 and glucose 11, pH 7.2) was K^+^-free PSS. The free Ca^2+^ concentration was approximately 70 nM as calculated using WEBMAXC STANDARD (http://www.stanford.edu/∼cpatton/webmaxc/webmaxcS.htm). NSCC currents were recorded with a ramp using a perforated whole-cell configuration with a holding potential of −60 mV. The ramp was performed over 500 ms from −80 to +60 mV.

### Statistical analysis

All data were expressed as the means ± standard deviation (SD). For all analyses, the evaluations were performed with Student’s *t* test using Origin 8.0 software (OriginLab, Northampton, MA, U.S.A.). *P*<0.05 was regarded as statistically significant.

## Results

### Coptisine relaxed high K^+^-induced pre-contraction in a dose-dependent manner

**Figure 1:**
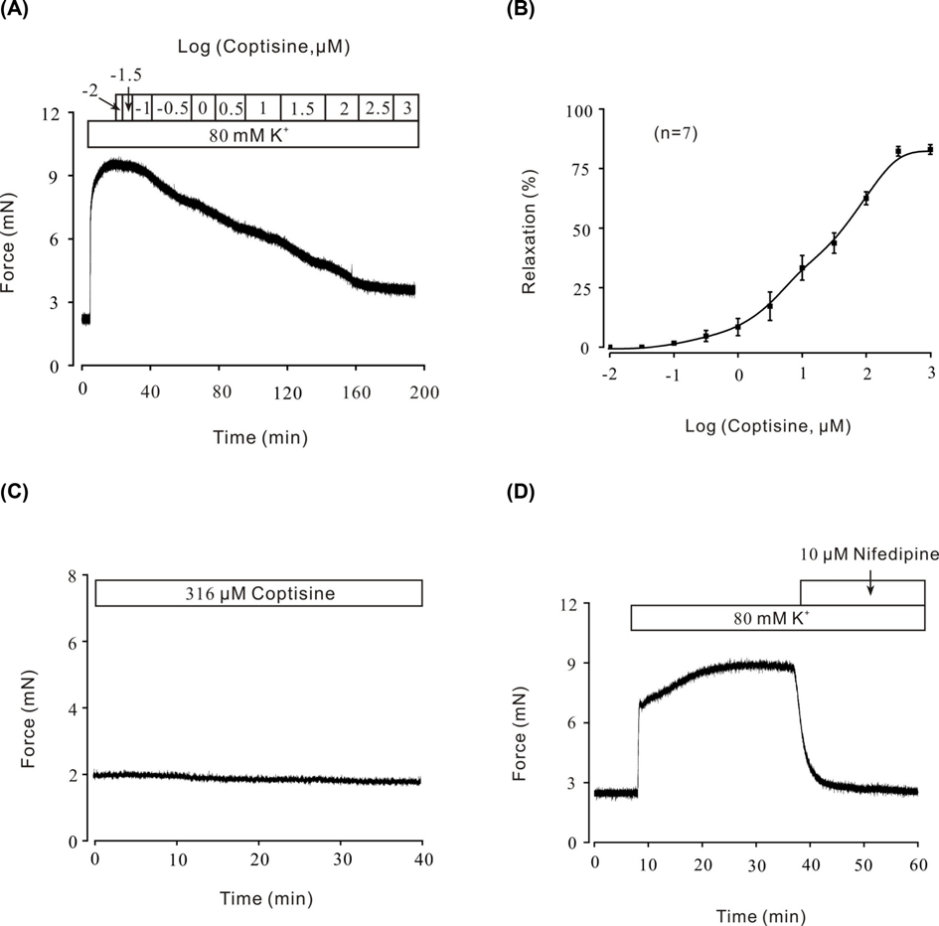
Relaxant effects of coptisine on high K^+^-induced pre-contraction (**A**) High K^+^ induced a steady-state contraction in a mouse tracheal ring, which was inhibited by coptisine in a dose-dependent manner. (**B**) Dose–relaxation curve of coptisine based on the results of seven different experiments (shown in A). (**C**) Coptisine had no effect on mouse tracheal ring of resting tension (*n*=6/6 mice). (**D**) High K^+^-induced pre-contraction was completely reversed by nifedipine (*n*=6/6 mice).

Previous research have demonstrated that high K^+^-induced smooth muscle contraction was mainly due to the depolarization of cell membrane, opening of VDLCC and influx of extracellular Ca^2+^, sequentially [20,21]. To explore the potential relaxant characteristic of coptisine, the dose–response curved was first calculated under presence of high K^+^. Our previous study has shown that high K^+^ could contract mouse tracheal ring gradually [16] and 80 mM K^+^ was applied to pre-contract ASM in this experiment. As shown in Figure 1A, the pre-contraction induced by high K^+^ (80 mM) was completely inhibited by coptisine (0.01–1000 μM) in a dose-dependent manner. According to the dose–contraction curve exhibited in Figure 1B, the maximal relaxation was calculated as 82.24 ± 4.94%. The half-maximal inhibition (IC_50_) was 45.76 ± 8.54 μM. The IC_75_ was 194.69 ± 12.38 μM (*n*=7/7 mice). Comparing with the relaxant characteristic of coptisine on pre-contracted mouse tracheal ring, 316 μM coptisine had no effect on resting mouse tracheal ring (Figure 1C). As shown in Figure 1D, 10 μM nifedipine, a selective blocker of VDLCCs [22], has a similar inhibitory on high K^+^‐induced steady state contraction in mouse tracheal rings (*n*=6/6 mice), which confirmed that the contraction was induced via the opening of VDLCCs. These results indicated that coptisine inhibited high K^+^-induced pre-contraction in a dose-dependent manner. Furthermore, the relaxant effect of nifedipine suggested that VDLCCs participated in high K^+^-induced contraction and also might be involved in coptisine-induced relaxation.

### Coptisine blocked high K^+^-evoked Ca^2+^ influx

**Figure 2:**
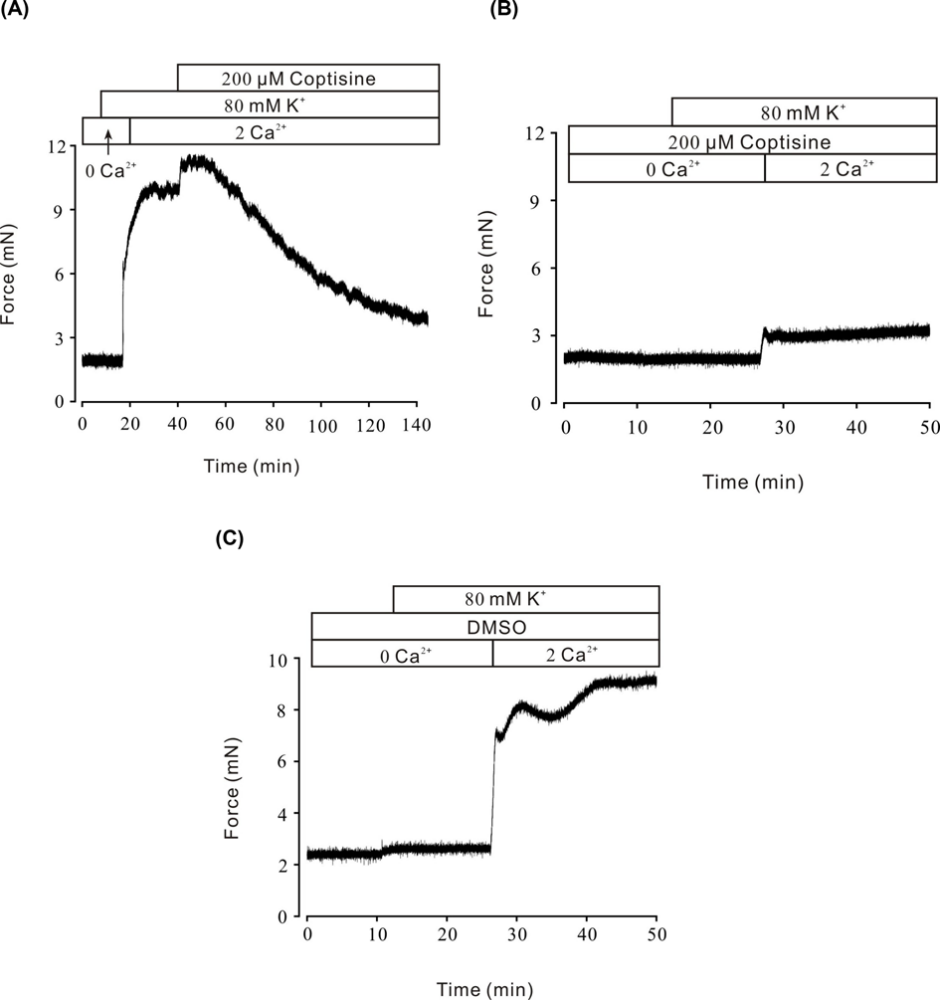
Coptisine block high K^+^-induced Ca^2+^ influx (**A**) In Ca^2+^-free medium (0 mM Ca^2+^ and 0.5 mM EGTA), high K^+^ did not show contractile effect in a mouse tracheal ring. After restoration of 2 mM Ca^2+^, a sustained contraction occurred, which was completely inhibited by 200 μM coptisine (*n*=7/7 mice). (**B**) In Ca^2+^-free solutions (0 mM Ca^2+^ and 0.5 mM EGTA), high K^+^ did not evoke contraction in 200 μM coptisine pretreated mouse tracheal ring. After restoration of 2 mM Ca^2+^, high K^+^ still could not evoke contraction (*n*=6/6 mice). (**C**) Under Ca^2+^-free conditions (0 mM Ca^2+^ and 0.5 mM EGTA), high K^+^ did not evoke contraction in coptisine’s solvent DMSO pretreated mouse tracheal ring. After restoration of 2 mM Ca^2+^, a sustained contraction occurred (*n*=6/6 mice).

The previous study on rat aortic ring indicated that coptisine could attenuate calcium release from the sarcoplasmic reticulum [23]. To further characterize the relaxant mechanism of coptisine shown in Figure 1, the following experiments were designed to explore whether calcium was also involved in coptisine-induced relaxation on mouse tracheal rings. In the Ca^2+^-free solution, high K^+^-induced contraction did not occur (Figure 2A), proving that calcium influx was necessary for VDLCC-induced contraction. Following Ca^2+^ restoration, the contraction that immediately evoked by high K^+^ was almost completely inhibited by 200 μM coptisine (Figure 2A) (*n*=7/7 mice). Meanwhile, high K^+^ failed to induce a pre-contraction under Ca^2+^-free conditions in the presence of 200 μM coptisine, and even after Ca^2+^ restoration, the ASM contraction was not obvious (*P*>0.05) (Figure 2B) (*n*=6/6 mice). It was supposed that VDLCCs has been blocked in coptisine pre-treated mouse tracheal ring, then high K^+^ could not evoke extracellular Ca^2+^ influx via blocked VDLCCs. As shown in Figure 2C, coptisine’s solvent, DMSO was applied as a negative control (*n*=6/6 mice). These data suggested that blocking high K^+^-induced Ca^2+^ influx was involved in the relaxant effects of coptisine.

### Coptisine blocked VDLCC currents

**Figure 3:**
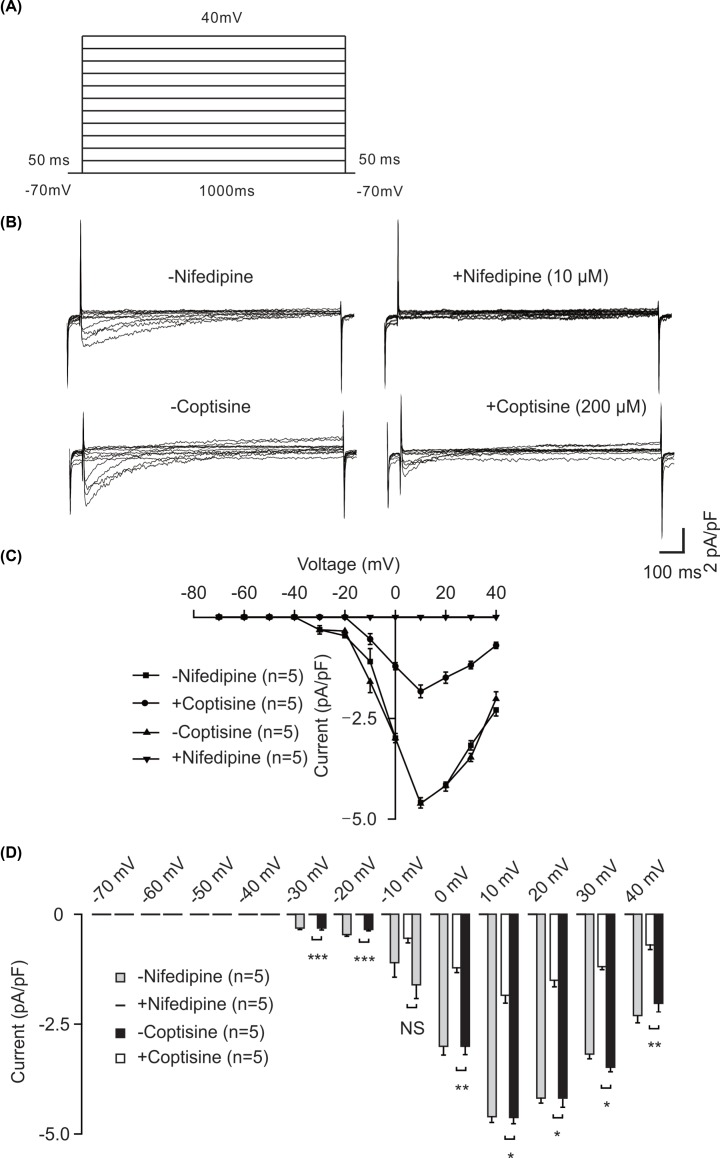
Coptisine blocked VDLCC currents (**A**) The VDLCC currents of single mouse ASM cells were calculated. (**B**) VDLCC currents were recorded following depolarization and were eliminated by coptisine or nifedipine. (**C**) The I–V relationship was constructed based on the results of five experiments. (**D**) The averaged current of NSCCs in the absence and presence of nifedipine or coptisine. NS, not significant, *, *P*<0.05, **, *P*<0.01, ***, *P*<0.001.

To further confirm the participation of VDLCCs in the ability of coptisine to relax ASM, particular VDLCC currents were measured using the whole-cell patch-clamp technique. As shown in Figure 3A, the currents were recorded with voltage steps from −70 to +40 mV. As a positive control, the currents were eliminated by the specific blocker nifedipine, indicating that VDLCC currents were recorded (Figure 3B, top). The currents were then inhibited by 200 μM coptisine (Figure 3B, bottom), which indicated that the effect of coptisine on VDLCC currents is similar to nifedipine. As a type of voltage-dependent channel, the current–voltage (*I–V*) curve of VDLCC was calculated to examine the voltage-dependent property (*n*=5/5 mice, Figure 3C). The averaged current of the VDLCCs in the absence and presence of nifedipine or coptisine are shown in Figure 3D. It was suggested that coptisine could inhibit VDLCC currents.

Taken together, these results demonstrated that coptisine could relax high K^+^-induced ASM contraction by blocking VDLCCs and then decreasing intracellular Ca^2+^.

### Coptisine relaxed ACh-induced pre-contraction in a dose-dependent manner

**Figure 4:**
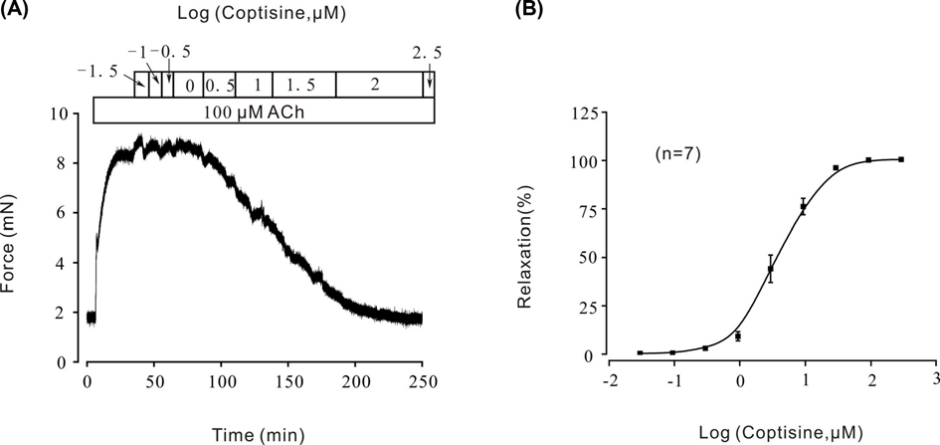
Coptisine inhibited ACh-induced pre-contraction in mouse tracheal rings (**A**) ACh induced a steady-state contraction in a mouse tracheal ring, which was inhibited by coptisine in a dose-dependent manner. (**B**) Dose–relaxation curve of coptisine based on the results of seven different experiments.

**Figure 5:**
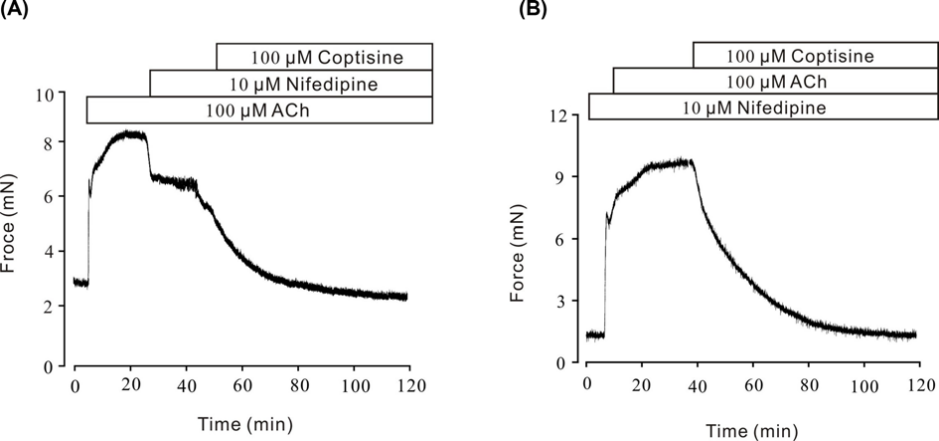
Coptisine relaxed ACh-induced contraction after excluding VDLCCs with nifedipine (**A**) Nifedipine partly reduced the ACh-induced contraction. The resistant contraction was inhibited by coptisine (*n*=7/7 mice). (**B**) ACh could induce contraction on nifedipine pretreated mouse tracheal ring, and which was inhibited by coptisine (*n*=6/6 mice).

The relaxation of smooth muscle is a complicated electrophysiological process and the collaboration of various ion channels is indispensable [24,25]. Thus, we wonder whether any other ion channels besides VDLCCs might also participate in coptisine-induced relaxation. ACh, a known muscarinic receptor agonist, which could evoke ASM contraction through both VDLCCs and NSCCs [26,27] was employed to pre-contract ASM. In our previous study, ACh could stimulate mouse tracheal contraction gradually [16] and the concentration of ACh was determined to be 100 μM to pretreat ASM in this experiment. Coptisine (0.0316–316 μM) was able to completely relax 100 μM ACh-induced pre-contraction in a dose-dependent manner (Figure 4A). Then the dose–response curve was calculated as shown in Figure 4B, The maximal relaxation was 100.00 ± 2.01%, and the IC_50_ was 4.02 ± 2.07 μM. IC_75_ was 10.51 ± 4.00 μM (*n*=7/7 mice). As shown in Figure 5, to isolate and identify the role of NSCCs, VDLCCs were excluded with the specific blocker nifedipine before or after ACh addition. In the presence of ACh, induced pre-contraction was partially reversed by 10 μM nifedipine (the average relaxation percentage was 36.29 ± 4.13%) (Figure 5A). Subsequently, 100 μM coptisine almost completely relaxed the remaining tension (Figure 5A, *n*=7/7 mice). In the presence of nifedipine, ACh-induced pre-contraction was also relaxed by 100 μM coptisine (Figure 5B, *n*=6/6 mice). These experiments indicated that besides VDLCCs, NSCCs could also be evoked by ACh and thus might involve in coptisine-induced relaxation.

### Coptisine blocked ACh-evoked Ca^2+^ influx

**Figure 6:**
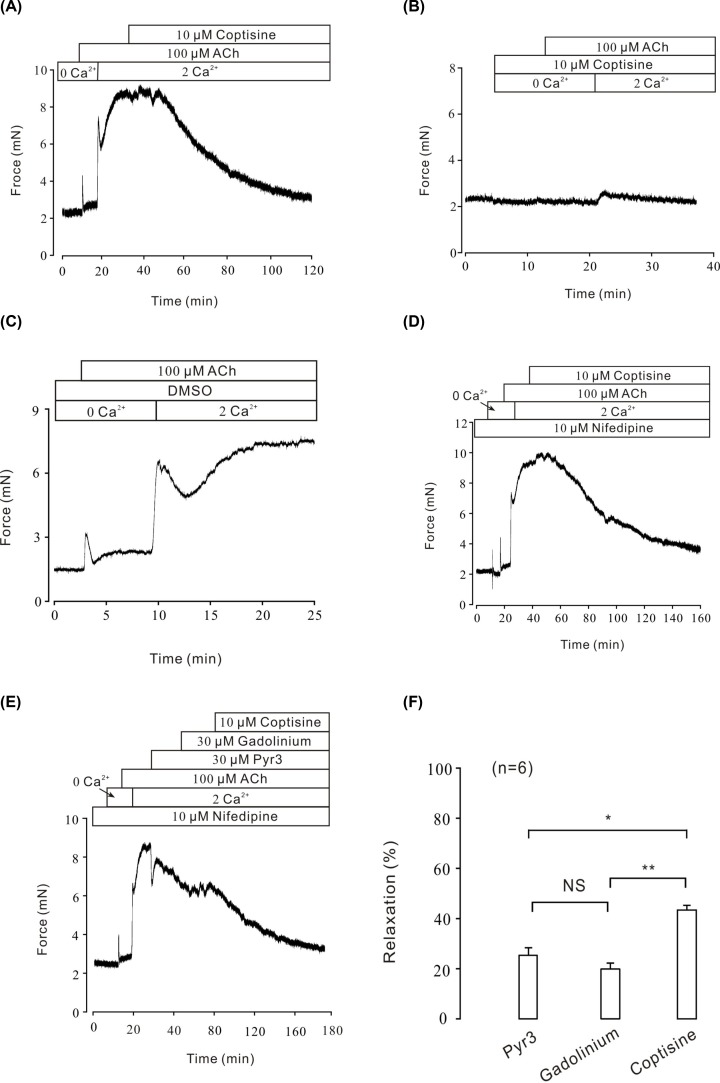
Coptisine blocked ACh-induced Ca^2+^ influx (**A**) In the presence of ACh and Ca^2+^-free solutions (0 mM Ca^2+^ + 0.5 mM EGTA), ACh induced a rapid, transient contraction. Following the restoration of 2 mM Ca^2+^, a strong, sustained contraction occurred, which was fully relaxed by coptisine (*n*=7/7 mice). (**B**) In the presence of coptisine, a Ca^2+^ restoration-induced contraction by ACh was not observed (*n*=6/6 mice). (**C**) In the presence of DMSO, the solvent of coptisine, and Ca^2+^-free conditions (0 mM Ca^2+^ + 0.5 mM EGTA), ACh induced a rapid, transient contraction. Following the restoration of 2 mM Ca^2+^, a strong, steady contraction occurred (*n*=6/6 mice). (**D**) In the presence of nifedipine and Ca^2+^-free medium (0 mM Ca^2+^ + 0.5 mM EGTA), ACh induced a rapid, transient contraction. Following the restoration of 2 mM Ca^2+^, a strong, steady contraction occurred, which was fully relaxed by coptisine (*n*=7/7 mice). (**E**) In the presence of nifedipine and Ca^2+^-free solutions (0 mM Ca^2+^ + 0.5 mM EGTA), ACh induced a rapid, transient contraction. Following the restoration of 2 mM Ca^2+^, a strong, sustained contraction occurred, which was relaxed by Pyr3, gadolinium and coptisine, sequentially. (**F**) The bar graph showed the average reduction in Pyr3, gadolinium and coptisine, respectively, from six experiments. NS, not significant; *, *P*<0.05, **, *P*<0.01.

Besides VDLCC, another possible source of Ca^2+^ entry during smooth muscle contraction is the NSCC permeable to external calcium ions [28]. To further extend the role of calcium in coptisine-evoked relaxation, Ca^2+^ entry through NSCCs was studied under ACh-induced pre-contraction. As shown in Figure 6A, in the presence of nifedipine (10 μM) under Ca^2+^-free condition, ACh induced a sharp contraction, indicating that ACh could transiently release Ca^2+^ from intracellular Ca^2+^ storage. Subsequently, the restoration of 2 mM Ca^2+^ triggered a steady contraction, which was completely eliminated by 10 μM coptisine (*n*=7/7 mice). However, in the presence of 10 μM coptisine, ACh failed to raise intracellular calcium. Even with the restoration of 2 mM Ca^2+^, sustained contraction also did not occur (Figure 6B). DMSO, the solution of coptisine was used as a negative control (Figure 6C). In order to isolate NSCCs evoked by ACh, VDLCC was blocked by nifedipine. As shown in Figure 6D, in Ca^2+^-free medium, intracellular Ca^2+^ transiently released after the addition of 100 μM ACh. With the restoration of 2 mM Ca^2+^, a sustained contraction was induced and subsequently reversed by 10 μM coptisine. These results indicated that Ca^2+^ influx played an important role in ACh-induced contraction. These data indicated that calcium mobilization via NSCC was both involved in ACh-induced contraction and coptisine-induced relaxation.

To further identify the specific components of NSCCs involved in coptisine-blocked Ca^2+^ influx, TRPC3 inhibitor Pyr3 [29,30] and gadolinium, which is a blocker of TRPC1, 3, 5, 6 and 7 [31] were employed sequentially. As shown in Figure 6E,F, in the presence of nifedipine, ACh induced a transient contraction under Ca^2+^-free conditions, which indicated that intracellular Ca^2+^ was transiently released after the addition of ACh. With the restoration of 2 mM Ca^2+^, a sustained contraction was induced by ACh and was partially reduced by 30 μM Pyr3 (the average relaxation percentage was 25.07 ± 6.94%), 30 μM gadolinium (the average relaxation percentage was 20.64 ± 4.87%), and finally almost completely eliminated by 10 μM coptisine (the average relaxation percentage was 43.26 ± 8.90%). Taken together, a critical molecular candidate for NSCC blocked by coptisine seems to be TRPC channels.

### Coptisine blocked NSCC currents

**Figure 7:**
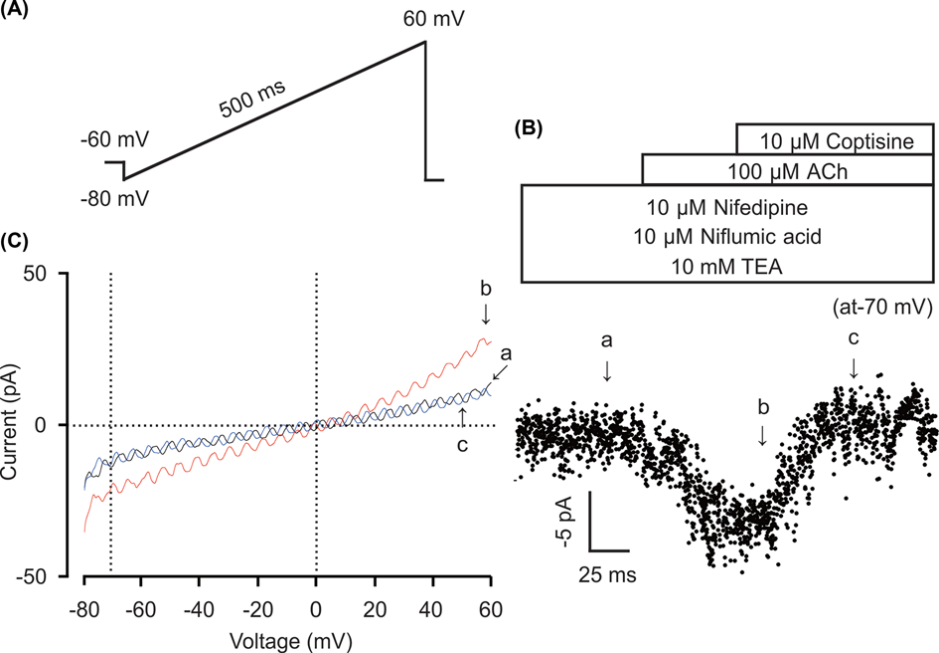
Coptisine blocked NSCC currents (**A**) The ramp clamp protocol was used to measure NSCC currents in single ASM cells. (**B**) *I–t* relationships were plotted at *V*_m_ = −70 mV. (**C**) The slope currents at times a, b, and c in (B).

To further test whether coptisine has some effects on NSCC currents, whole-cell patch-clamp was employed to measure ACh-induced NSCC currents with or without coptisine (Figure 7). The NSCC current showed a ramp from −80 to +60 mV (Figure 7A). To block currents from VDLCCs, Cl^−^ channels and K^+^ channels, nifedipine, NA and TEA were applied respectively. Thus, the residual current was ACh-induced NSCC current. As shown in Figure 7B, NSCC currents could be completely blocked by 10 μM coptisine (*n*=6/6 mice). Three representative ramp current traces at time points a, b and c are shown in Figure 7C. Taken together, these results indicate that coptisine can inhibit ACh-induced NSCC currents.

### Na^+^/Ca^2+^ exchangers did not involve in coptisine-induced relaxation

**Figure 8:**
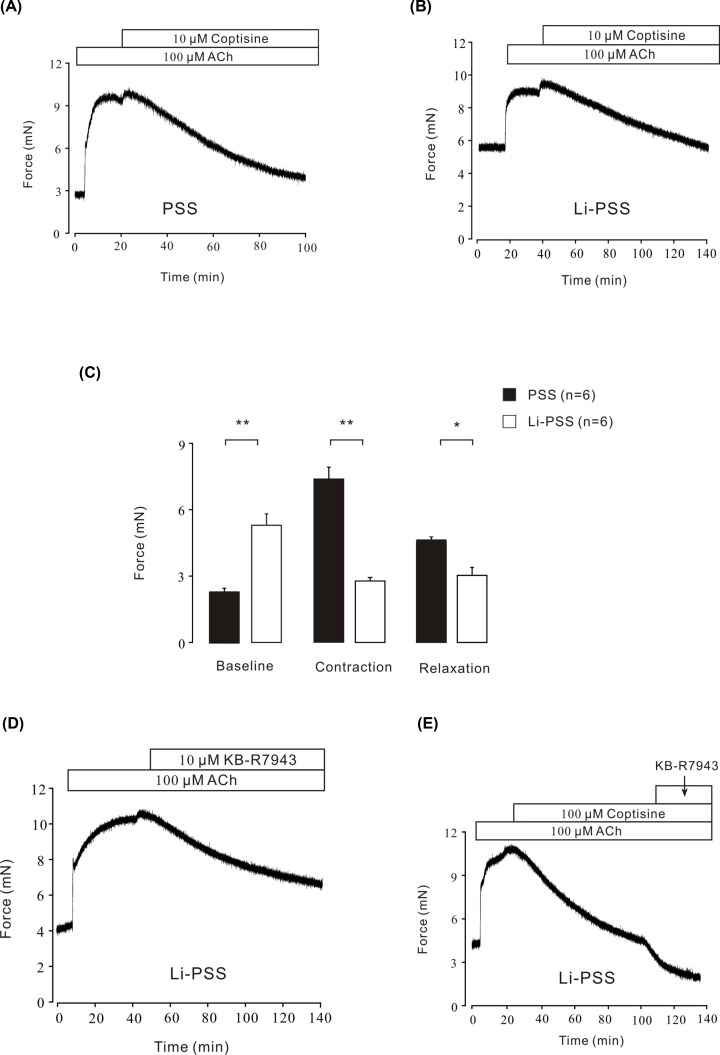
Coptisine could not inhibit NCXs (**A**) Coptisine reversed ACh-induced contraction under PSS condition (*n*=6/6 mice). (**B**) Coptisine reversed ACh-induced contraction under Li-PSS condition (*n*=6/6 mice). (**C**) The bar graph showed the comparisons of forces at the baseline, contraction, relaxation from six experiments, respectively. *, *P*<0.05, **, *P*<0.01. (**D**) In Li-PSS solution, KB-R7943 partially reversed ACh-induced contraction (*n*=6/6 mice). (**E**) In Li-PSS medium, after coptisine reversed ACh-induced contraction, KB-R7943 could subsequently relax the mouse tracheal ring lower than the baseline (*n*=6/6 mice).

Besides VDLCC_S_ and NSCC_S_, Na^+^/Ca^2+^ exchangers (NCX) also play a critical role in the intake of Ca^2+^ by cells in smooth muscle [32]. To explore the role of NCX in Ca^2+^ influx blocked by coptisine, Li-PSS without sodium was applied instead of PSS to evoke Ca^2+^ influx via NCX. As shown in the Figure 8B, it turned out that under the condition of Li-PSS, ACh-induced a prominent contraction with an obviously higher baseline compared with PSS condition (Figure 8A), which indicated that NCX might be switched to a ‘Ca^2+^ influx/Na^+^ outflow’ mode to increase intracellular Ca^2+^. Following addition of 10 μM coptisine, the contraction was potently attenuated to the base line (Figure 8A–C). Furthermore, KB-R7943, a specific NCX blocker [33,34] was applied to identify the role of NCX in coptisine-induced relaxation. As shown in Figure 8D, KB-R7943 could reverse the ACh-induced contraction (the average relaxation percentage was 54.16 ± 3.62%) in Li-PSS solution. However, in the presence of 100 μM coptisine, KB-R7943 could continuously relax ACh-induced contraction ever lower than the baseline. The data indicated that NCX was probably not blocked in coptisine-induced relaxation.

## Discussion

Recently, various studies focused on TCM for a safer and milder treatment of pulmonary disease. A plurality of the investigated natural herbal extracts or single compound exert their relaxant effect on precontracted airway muscle [7,8]. Coptisine is a natural compound which displays a broad range of pharmacological actions [11–15]. Previous studies have evaluated the vasorelaxant effects of coptisine on isolated rat aortic rings [23], which shed light on the possible mechanism of coptisine’s action on abnormal contracted mouse tracheal rings. In current study, we investigated the relaxant effects of coptisine in agonist-triggered ASM contraction and the underlying mechanisms. We first examined whether coptisine could relax abnormal contracted mouse tracheal rings. It turned out that coptisine could inhibit contractile effect of mouse tracheal rings induced by high K^+^ or ACh in a concentration-dependent way. VDLCCs and NSCCs are two categories of voltage-dependent and receptor-operated channel candidates which are critical for intracellular and extracellular calcium mobilization in ASM contraction [26,27,35,36]. To investigate the relaxant effect of coptisine, calcium mobilization in ASM were further calculated. The results indicated that calcium oscillation played an important role in coptisine-induced relaxation by blocking VDLCCs and NSCCs, especially TRPCs. However, it should be noted that except for VDLCC and NSCC, NCX did not play a role in coptisine-induced relaxation [32]. To further identified the roles of VDLCCs and NSCCs in coptisine-induced relaxation, VDLCC or NSCC currents were measured. Coptisine was found to eliminate both VDLCC and NSCC currents.

In conclusion, coptisine is an important ingredient of TCM which has shown various medical properties. Through current study, its pharmacological characteristic has been extended to relaxant effect on mouse ASM and the underlying molecular mechanism has been clarified. Further fundamental studies and clinical trials are required to explore more therapeutic properties of TCM including coptisine and explain the underlying molecular mechanism with modern scientific language.

## Conclusion

In summary, our research indicated that pretreatment of mouse ASM with high K^+^ or ACh could be relaxed by coptisine through blocking VDLCCs and NSCCs then inhibiting calcium influx. Our research work provided evidence that coptisine might have potential therapeutic value for the treatment of pulmonary disease associated with abnormal ASM contraction.

## Abbreviations

ACh: acetylcholine chloride
ASM: airway smooth muscle
BSA: bovine serum albumin
DMSO: dimethyl sulfoxide
IC_50_: half-maximal inhibition
NA: niflumic acid
NCX: Na^+^/Ca^2+^ exchanger
NSCC: non-selective cation channel
PSS: physiological salts solution
Pyr3: pyrazole 3
TCM: traditional Chinese medicine
TEA: tetraethylammonium chloride
VDLCC: voltage-dependent L-type Ca^2+^ channel
